# Stability of empathy among undergraduate medical students: A longitudinal study at one UK medical school

**DOI:** 10.1186/1472-6920-11-90

**Published:** 2011-10-25

**Authors:** Thelma A Quince, Richard A Parker, Diana F Wood, John A Benson

**Affiliations:** 1 General Practice and Primary Care Research Unit, Department of Public Health and Primary Care, Institute of Public Health, Forvie Site, Robinson Way, Cambridge CB2 0SR, UK; 2 University of Cambridge School of Clinical Medicine, Box 111 Addenbrookes Hospital, Hills Road, Cambridge CB2 2SP, UK

## Abstract

**Background:**

Empathy is important to patient care. The prevailing view is that empathy declines during university medical education. The significance of that decline has been debated.

This paper reports the findings in respect of two questions relating to university medical education:

1. Do men and women medical students differ in empathy?

2. Does empathy change amongst men and women over time?

**Methods:**

The medical course at the University of Cambridge comprises two components: Core Science (Years 1-3) and Clinical (Years 4-6). Data were obtained from repeated questionnaire surveys of medical students from each component over a period of four years: 2007-2010. Participation in the study was voluntary.

Empathy was measured using two subscales of the Interpersonal Reactivity Index: IRI-EC (affective empathy) and IRI-PT (cognitive empathy). We analysed data separately for men and women from the Core Science and Clinical components. We undertook missing value analyses using logistic regression separately, for each measure of empathy, to examine non-response bias. We used Student's t-tests to examine gender differences and linear mixed effects regression analyses to examine changes over time. To assess the influence of outliers, we repeated the linear mixed effects regression analyses having excluded them.

**Results:**

Women displayed statistically significant higher mean scores than men for affective empathy in all 6 years of medical training and for cognitive empathy in 4 out of 6 years - Years 1 and 2 (Core Science component) and Years 4 and 5 (Clinical component).

Amongst men, affective empathy declined slightly during both Core Science and Clinical components. Although statistically significant, both of these changes were extremely small. Cognitive empathy was unchanged during either component. Amongst women, neither affective empathy nor cognitive empathy changed during either component of the course.

Analysis following removal of outliers showed a statistically significant slight increase in men's cognitive empathy during the Core Science component and slight decline in women's affective empathy during the Clinical component. Again, although statistically significant, these changes were extremely small and do not influence the study's overall conclusions.

**Conclusions:**

Amongst medical students at the University of Cambridge, women are more empathetic than men (a generally observed phenomenon). Men's affective empathy declined slightly across the course overall, whilst women's affective empathy showed no change. Neither men nor women showed any change in cognitive empathy during the course. Although statistically significant, the size of such changes as occurred makes their practical significance questionable. Neither men nor women appear to become meaningfully less empathetic during their medical education at the University of Cambridge.

## Background

In recent years much research into patient care and medical education has focused on empathy. Empathy is seen as one of the personal qualities that defines professionalism in medicine [1,2] and as a pre-requisite for "patient centred" care [3]. Patients have been found to report higher levels of satisfaction, comfort and self-efficacy when doctors are more empathetic [4-6]. Empathy facilitates the development of trust and openness, enables more accurate diagnosis and possibly fosters greater adherence to treatment regimes [7,8]. Being in receipt of physician empathy may have a direct influence on clinical outcomes [9].

Empathy in the doctor-patient relationship may also benefit the doctor [10]. Displaying empathy may enhance job satisfaction by making medicine less frustrating [11]. Diminished empathy has been found to be associated with higher levels of physician burnout, which in turn may be associated with increased likelihood of perceived medical error, [12-14] although the causality in this relationship remains unclear.

Empathy is often poorly defined in medical education research [15]. However across differing fields of study such as psychology, child and adolescent development and criminology there is a broad consensus that empathy is a multidimensional construct comprising:

a] an affective capacity to be sensitive to and concerned for another person [16-19].

b] a cognitive capacity to understand and appreciate the perspective of another person. In the medical context it has been suggested that the cognitive dimension extends also to the ability to communicate that understanding [20].

The prevailing view is that empathy declines during university medical education [21-25]. However this view remains open to question. Firstly, many studies suggesting the decline pre-date changes affecting medical education which may enhance the development of empathy, such as integration between preclinical and clinical work, early patient contact and communication skills teaching [26]. Secondly, although some longitudinal work has been undertaken [25,27], many studies are cross sectional, preventing analysis of change in individuals over time. Thirdly, a reported overall trend may mask different or even opposing trends displayed by different subgroups. For example, despite evidence that women display higher levels of empathy, [27-31] few studies distinguish between results for men and women. Fourthly the stage of medical education at which this decline occurs appears to vary: some studies suggest it is most pronounced in the later years of medical education [27], whilst others suggest that decline occurs in the early years [32]. Finally, although some reported declines in medical student empathy have been statistically significant, the practical significance of those declines remains open to debate [33]. Some scales used to measure empathy have been used in the wider population, but none has an agreed range in which empathy might be scaled for medical practice [16,25]. This is especially the case for medical students and young doctors for whom norms derived from the general population are of questionable relevance given evidence that empathy differs with age [34,35].

The School of Clinical Medicine at the University of Cambridge is engaged in the study of factors in undergraduate medical education which influence the quality of patient care provided by students in their subsequent medical practice. We regard empathy as one such factor. In October 2007, we began a longitudinal study involving all students coming to Cambridge to study medicine. This paper reports the findings in respect of two questions relating to university medical education:

1. Do men and women medical students differ in empathy?

2. Does empathy change amongst men and women over time?

## Methods

### Participants

**Figure 1:**
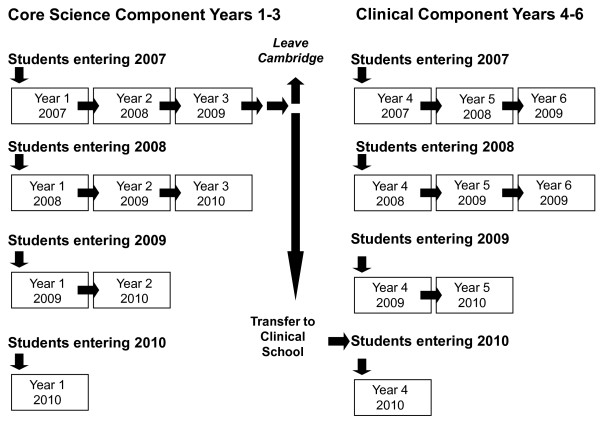
**Components of the Cambridge course and pattern of participation in the study**.

The course at Cambridge comprises: a Core Science component (Years 1-3) during which students learn core medical science (Year 3 comprising options which may not be medicine or science-related) with a small element of clinical experience; and a Clinical component (Years 4-6) during which students learn in a clinical environment (Figure 1). Between 270 and 290 students, typically aged 18 years, enter the Core Science component. At the end of Year 3, approximately half of these continue into the Clinical component, joined by a small number of students from other universities. From September 2007, all students entering Years 1 and 4 (the first years of the Core Science and Clinical components respectively) were invited to take part in a longitudinal study comprising an annual questionnaire survey. To date, participants comprise students entering the Core Science and Clinical components in 2007, 2008, 2009 and 2010. Students entering the Clinical Component in 2010 comprised those students who had entered the Core Science component in 2007, who remained in Cambridge.

### Measures

We regarded empathy as comprising affective and cognitive elements requiring separate measurement. Entrants to the Core Science component are typically aged 18 and hence have limited clinical experience, so we chose to measure empathy with a generic instrument, rather than one designed specifically for medical personnel: Davis's Interpersonal Reactivity Index (IRI) [16]. The IRI is a self-report measure comprising 28 mixed positive and negative statements, with response options ranging from "Does describe me very well" to "Does not describe me very well", rated 0 to 4. It comprises 4 subscales, each with 7 items, of which two: Empathetic Concern (IRI-EC) and Perspective Taking (IRI-PT) have been used extensively in the fields of adolescent development, criminology and medical education. IRI-EC measures the affective dimension of empathy and IRI-PT the cognitive dimension. Higher scores indicate greater empathy. Reviews of these subscales in the fields of both medicine and criminology indicate good psychometric properties, with reliability, as measured by Cronbach's α, typically greater than 0.75 [18,36,37].

### Procedures

Participation was voluntary with students completing the questionnaire in their own time. A paper-based questionnaire was used for all students in 2007 and 2008, and for Clinical students in 2009. An on-line questionnaire was used for Core Science students in 2009 and for all students in 2010 (Figure 1). Questionnaires were distributed to students during the first week of each new academic year, with the exception of Clinical students in Year 6, who were asked to participate towards the end of their training. Questionnaires were labelled only by study number, with names unavailable to the research team. A study data manager (who had no access to results) sent one reminder to students after 2 weeks. A small prize to reward participation was awarded annually by lottery to a small number of participants. The study received approval from the University of Cambridge Psychology Ethics Committee.

### Analysis

#### Overall approach

Data for each subscale of the IRI was collected repeatedly overtime and is therefore longitudinal. Only half of the Core Science students present in Years 1-3 remain in Cambridge for Years 4-6, yielding small numbers for analysis across all 6 years (Figure 1). For this reason, we analysed data separately for students from the Core Science component (Years 1-3) and students from the Clinical component (Years 4-6). For all analyses, statistical significance was set at the 5% level (p < 0.05).

#### Non-response bias

In order to assess the presence of non-response bias, we undertook missing value analyses using logistic regression separately for men and women, for each measure of empathy. In all models, year of course entry was included as an explanatory factor variable, and hence all the results for missing data analysis were adjusted for student cohort. Outcome variables were missing values at Years 2 and 3 for students entering the Core Science component and missing values at Years 5 and 6 for students entering the Clinical component. All students entering either component in years 2007, 2008 and 2009 and completing a questionnaire on entry were included. Students entering either component in 2010 were excluded because they had only one year of data.

#### Empathy and Gender

To determine whether men and women differed with respect to IRI-EC and IRI-PT during each component of the course, (Core Science and Clinical) we conducted Student's t-tests comparing scores for men and women at each time point and calculated effect size scores (Cohen's d) for the difference between men and women.

#### Change in empathy

To examine changes in empathy over time among students within each component of the course (Core Science and Clinical), we undertook regression analyses, using linear mixed effects regression models, for men and women separately, for the outcome variables of IRI-EC and IRI-PT. In order to adjust for any effect of student year of entry, we included it as an explanatory factor variable in the regression models. Individual students were modeled as random effects, assuming a general correlation structure (i.e. unstructured within-student correlation). All students providing data on IRI-EC or IRI-PT for at least one time point were included.

In order to explore the extent to which outliers influenced our results and thereby test the robustness of models, we applied a sensitivity analysis to the outcome variables, (IRI-EC and IRI-PT) by repeating the regression analyses after removing outliers more than 3 standard deviations away from the mean.

#### Inferences between Core Science and Clinical Components of the Course

In order to judge whether we could make inferences about trends carrying through from the Core Science component of the course to the Clinical component, we used ANOVA (Bonferroni post hoc tests) to compare separately students entering the Clinical component in 2007, 2008 and 2009 with those who entered in 2010, since the majority of the latter had entered the Core Science component of the course in 2007 (Figure 1).

## Results

### Respondents and non-response bias

Table 1 shows the number of entrants to the Core Science and Clinical components of the course for each year of entry (2007-2010) together with those who participated each year within each component. Table 2 shows the missing value analysis. For students in the Core Science component of the course this indicated that scores for IRI-EC and IRI-PT on entry at Year 1 did not significantly predict non-response in Years 2 and 3 (Table 2). Similarly, for students in the Clinical component of the course indicated that scores for IRI-EC and IRI-PT on entry at Year 4 did not significantly predict non-response in Years 5 and 6 (Table 2). Odds ratio are presented with 95% confidence intervals and p-values.

**Table 1:** Number of students taking part in the survey at each year for each component of the course

Core Science Component				
		Year of Component		
		Year 1	Year 2	Year 3
Year of entry	Total number of entrants	Participants (as % of total entrants) *(Women as % of participants)*	Participants (as % of total entrants) *(Women as % of participants)*	Participants (as % of total entrants) *(Women as % of participants)*

2007	266	183 (68.8%) *(50.8%)*	144 (54.1%) *(52.4%)*	121 (45.5%) *(54.2%)*
2008	283	137 (48.5%) *(58.4%)*	87 (30.7%) *(60.9%)*	78 (27.6%) *(60.3%)*
2009	281	155 (55.1%) *(53.2%)*	94 (33.4%) *(53.2%)*	
2010	282	189 (67.0%) *(50.3%)*		

Total Core Science entrants	1111	664 *(52.8%)*	325 *(54.9%)*	199 *(56.6%)*

**Clinical Component**				

	Year of Component			
		Year 4	Year 5	Year 6
Year of entry	Total number of entrants	Participants (as % of total entrants) *(Women as % of participants)*	Participants (as % of total entrants) *(Women as % of participants)*	Participants (as % of total entrants) *(Women as % of participants)*

2007	135	104 (77.0%) *(63.5%)*	82 (60.7%) *(67.1%)*	76 (56.3%) *(67.1%)*
2008	135	101 (74.8%) *(54.5%)*	70 (51.9%) *(58.6%)*	57 (42.2%) *(63.2%)*
2009	135	70 (51.9%) *(46.4%)*	47 (34.8%) *(45.7%)*	
2010	137	69 (50.4%) *(47.8%)*		

Total Clinical entrants	542	343 *(54.5%)*	199 *(59.1%)*	133 *(65.4%)*

**Table 2:** Missing value analysis: Regression results presented as odds ratios, 95% confidence intervals, and p-values.

	Men	Women
**Core Science component students**		
**Empathetic Concern**		
odds ratio	0.998	1.033
(95%CI)	(0.932 to 1.069)	(0.960 to 1.111)
p values	p = 0.96	p = 0.38
**Perspective Taking**		
odds ratio	0.988	1.001
(95%CI)	(0.923 to 1.057)	(0.936 to 1.070)
p values	p = 0.73	p = 0.99

**Clinical component students**		
**Empathetic Concern**		
odds ratio	1.036	0.999
(95%CI)	(0.955 to 1.123)	(0.913 to 1.092)
p values	p = 0.40	p = 0.98
**Perspective Taking**		
odds ratio	1.024	0.935
(95%CI)	(0.948 to 1.107)	(0.867 to 1.008)
p values	p = 0.54	p = 0.08

### Empathy and Gender

Table 3 shows the mean scores for both measures of empathy for all men and women participating, results of t-tests and effect size scores (Cohen's d).

**Table 3:** Results of gender comparison of IRI-EC and IRI-PT mean scores.

Empathetic Concern				
**Core Science component students**				
Year of course		Year 1	Year 2	Year 3

**Men**		n = 309	n = 145	n = 86
	Mean (SD)	19.43 (4.02)	18.13 (4.87)	18.77 (4.15)
**Women**		n = 346	n = 175	n = 112
	Mean (SD)	21.07 (3.76)	21.24 (3.71)	20.84 (3.78)
		t = 5.379 p < 0.001	t = 6.479 p < 0.001	t = 3.665 p < 0.001
	Cohen's d	0.42	0.73	0.53

**Clinical component students**				
Year of course		Year 4	Year 5	Year 6

**Men**		n = 154	n = 81	n = 45
	Mean(SD)	19.47 (4.09)	18.89 (4.54)	19.02 (4.12)
**Women**		n = 182	n = 115	n = 87
	Mean(SD)	21.58 (3.54)	21.66 (3.28)	21.16 (3.48)
		t = 5.069 p < 0.001	t = 4.699 p < 0.001	t = 3.138 p = 0.002
	Cohen's d	0.56	0.72	0.58

**Perspective Taking**				

**Core Science component students**				
Year of Course		Year 1	Year 2	Year 3

**Men**		n = 310	n = 145	n = 86
	Mean (SD)	18.05 (4.21)	17.60 (4.91)	18.37 (3.97)
**Women**		n = 346	n = 175	n = 112
	Mean (SD)	19.37 (3.98)	19.64 (4.11)	19.44 (4.53)
		t = 4.133 p < 0.001	t = 4.043 p < 0.001	t = 1.730 p = 0.085
	Cohen's d	0.32	0.47	0.25

**Clinical component students**				
Year of Course		Year 4	Year 5	Year 6

**Men**		n = 155	n = 81	n = 45
	Mean (SD)	17.90 (4.21)	17.75 (4.53)	18.24 (4.69)
**Women**		n = 183	n = 114	n = 87
	Mean (SD)	19.22 (4.20)	19.68 (3.75)	19.03 (4.21)
		t = 2.865 p < 0.004	t = 3.236 p < 0.001	t = 0.984 p = 0.327
	Cohen's d	0.31	0.47	0.18

#### Core Science component students

(Table 3) For IRI-EC, gender differences were statistically significant at each year of the course, with differences in mean scores for men and women ranging from 0.42 to 0.73 of a standard deviation unit. For IRI-PT, gender differences were statistically significant in Years 1 and 2, but not in Year 3, with differences in mean scores for men and women of 0.25 to 0.47 of a standard deviation unit in Years 1 and 2 respectively.

#### Clinical Component Students

(Table 3) For IRI-EC, gender differences were statistically significant at each stage of the course, with differences in the mean scores for men and women ranging from 0.56 to 0.72 of a standard deviation unit. For IRI-PT, gender differences were statistically significant Years 4 and 5, but not in Year 6, with differences in the mean scores for men and women of 0.31 and 0.47 of a standard deviation unit in Years 4 and 5 respectively.

### Change in empathy

Table 4 shows the results of linear mixed effects regression analyses, conducted separately for men and women, examining changes over time for the outcomes IRI-EC and IRI-PT. Time coefficients (i.e. mean differences between years of course) are presented with 95% confidence intervals.

**Table 4:** Time coefficients resulting from regression analyses for the outcome variables of IRI-EC and IRI-PT, controlling for student year of entry, presented as time coefficients with 95% confidence intervals

	Men	Women
**Core Science component students**		
**Empathetic Concern**		
Time coefficients (95% CI)	-0.53 (-0.87 to -0.20)	-0.12 (-0.39 to 0.14)
**Perspective Taking**		
Time coefficients (95% CI)	0.35 (-0.006 to 0.71)‡	-0.03 (-0.37 to 0.30)
**Clinical component students**		
**Empathetic Concern**		
Time coefficients (95% CI)	-0.46 (-0.89 to -0.02)	-0.27 (-0.55 to 0.01)*
**Perspective Taking**		
Time coefficients (95% CI)	0.14 (-0.31 to 0.60)	-0.13 (-0.35 to 0.09)

Results were similar and conclusions unchanged after removing outliers more than 3 SD away from the mean, except where indicated.

‡ Time coefficient was 0.38 (95% CI 0.03 to 0.74) after removing outliers more than 3 SD below the mean.

* Time coefficient was -0.30 (95% CI -0.57 to -0.04) after removing outliers more than 3 SD below the mean.

#### Primary analyses

##### Core Science component students

There was a small, significant decline in IRI-EC amongst men, but no significant change amongst women. No significant change in IRI-PT was found amongst either men or women (Table 4).

##### Clinical component students

There was a small, significant decline in IRI-EC amongst men, but no significant change amongst women. No significant change in IRI-PT was found amongst either men or women (Table 4).

#### Sensitivity analyses (removal of outliers more than 3 standard deviations away from the mean)

Analysis with outliers excluded indicated a small, significant increase in IRI-PT amongst Core Science men and a small, significant decline in IRI-EC amongst women in the Clinical component (Table 4).

The changes in empathy over time were statistically significant among men, however the magnitude of the changes indicated by the regression coefficients were extremely small (Table 4). The largest regression coefficient seen was -0.53, where scales for both IRI-EC and IRI-PT range from zero to 28.

### Inferences between Core Science and Clinical Components of the Course

To explore the view that trajectories observed among students in the Clinical component of the course could be repeated by students from the Core Science component when they enter the Clinical component we used ANOVA to compare scores for IRI-EC and IRI-PT recorded by students entering the Clinical component (Year 4) in September 2007 and 2008 and 2009 with those who entered the Clinical component (Year 4) in 2010. Bonferroni post hoc tests show no significant differences between students entering the Clinical component in 2010 and students entering in any of the earlier years. This was true for both measures of empathy and for both men and women (Table 5).

**Table 5:** Comparison of mean scores for IRI-EC and IRI-PT of students entering the Clinical component of the course (Year 4) in 2007, 2008 and 2009 with those entering in 2010, by gender

	Empathetic Concern	
Students entering Clinical component (Year 4)	**Men**	**Women**
2010	n = 35	n = 34
Mean (SD)	19.37 (4.28)	21.48 (4.04)
2007	n = 37	n = 64
Mean difference from 2010 (95% CI)	-0.169 (-2.76 to 2.43)	-0.620 (-2.09 to 1.96)
2008	n = 46	n = 55
Mean difference from 2010 (95% CI)	0.263 (-2.21 to 2.73)	-0.645 (-2.73 to 1.44)
2009	n = 36	n = 31
Mean difference from 2010 (95% CI)	-0.573 (-3.19 to 2.04)	0.711 (-1.65 to 3.07)

	**Perspective Taking**	

Students entering Clinical component (Year 4)	**Men**	**Women**
2010	n = 35	n = 34
Mean (SD)	18.11 (4.05)	18.76 (4.36)
2007	n = 37	n = 64
Mean difference from 2010 (95% CI)	0.736 (-1.94 to 3.41)	-0.992 (-3.40 to 1.42)
2008	n = 46	n = 54
Mean difference from 2010 (95% CI)	0.093 (-2.45 to 2.63)	-0.533 (-3.01 to 1.94)
2009	n = 37	n = 31
Mean difference from 2010 (95% CI)	0.033 (-2.64 to 2.71)	0.274 (-2.54 to 3.08)

(Bonferroni post hoc test results)

## Discussion

Our study obtained data on empathy from students in all 6 years of medical training. Between 55% and 78% of each cohort in the Core Science component of the course participated at some point in the study. The comparable figures for the Clinical component were 50% to 82%. These figures, allied with missing data analyses indicating that initial scores did not predict later non-response, would support the view that the findings can reasonably be generalized to the population of medical students at the University of Cambridge.

We found statistically significant gender differences in affective empathy at all 6 years of medical training and in cognitive empathy for 4 years. These findings support those of other studies among different populations, using a range of instruments [24,25,27,32].

Differences in mean scores between men and women were larger than any of the changes in mean scores between different stages of the course. Gender differences in IRI-EC ranged from 1.64 to 3.11 and for IRI-PT from 0.79 to 2.04. Differences in mean scores at different stages of the course were generally less than 1.

We found that with time, affective empathy declined on average for men and sensitivity analysis (removal of outliers) revealed that women's affective empathy declined in the Clinical component of the course. Amongst women in the Core science component of the course affective empathy remained constant on average. There were no significant changes in cognitive empathy amongst women or amongst Clinical men. Sensitivity analysis (removal of outliers) revealed that in the Core Science component of the course men's cognitive empathy increased. However, although these changes were statistically significant, regression coefficients indicated that they were extremely small and therefore of questionable practical significance.

By using the generic IRI, we were able to differentiate between affective and cognitive dimensions of empathy. This is at variance with more recent studies of empathy using the Jefferson Scale of Physician Empathy (JSPE) [9,22,24,27,30-32,38-40]. However our approach enables us to set in context empathy scores recorded by our medical students. The mean scores recorded by students in our study for both IRI-EC and IRI-PT (at Year 1, males IRI-EC 19.43, IRI-PT 18.05, females IRI-EC 21.07, IRI-PT 19.37) are similar to those recorded by medical students in other studies [21,28,38,39]. The scores also resemble those obtained from studies of other undergraduate student populations [16,21,41,42].

However, apart from general notions of more empathy being better for patient care there are no benchmarks for medical student empathy. Further, comparisons of scores for a generic instrument such as the IRI recorded by medical students with other populations do little to inform medical education. Given its widespread use in a medical context, perhaps it is now time for benchmarks for medical students to be established for the JSPE, which also take account of possible differences in age and culture [39,40].

Our findings would suggest that any changes observed in either affective or cognitive empathy amongst Cambridge medical students were small and of limited practical significance. This supports the view expressed by Colliver [33] in the recent debate about decline in medical student empathy [43-45].

The investigation reported here is limited by being based on one UK medical school, providing a "traditional" course. The voluntary nature of the survey meant that initial response rates were variable (Table 1). Students entering in 2007 and 2008 have had the opportunity to participate on 3 occasions. Of these, 29% of Core Science students and 45% of Clinical students have done so. Nevertheless, the missing value analysis supports the view that those continuing to participate could be considered representative of all student entrants in their year group and that continued participation was not influenced by levels of either affective or cognitive empathy recorded at the beginning of participation. However, since the missing value analysis is based only on initial values for affective and cognitive empathy, we cannot completely exclude the possibility of an association between an unobserved change in either affective or cognitive empathy and the missing values.

Although the IRI enables measurement of different dimensions of empathy it is nonetheless a self-report instrument and we cannot predict the extent to which reported levels of empathy are reflected in the actual behaviours of our students or influenced by socially desirable responses.

The results of this study highlight further research questions. For example how far does the gender difference in affective empathy persist after qualification? Is it a reflection of some innate difference which may have implications for selection?

## Conclusions

Our study suggests that compared to women, men recorded lower levels of affective empathy throughout their course and lower levels of cognitive empathy for part of their medical course. For each component of the course, on average, men's affective empathy declined by very small amounts over time. Women's affective empathy appeared to be more stable. Although mean changes in affective empathy for men in each component of the course were statistically significant, they were small enough to be of questionable practical significance. There were no declines in cognitive empathy for any group of students. Amongst medical students at this University, men appear to be less empathetic than women (a generally observed phenomenon), but neither men nor women appear to become meaningfully less empathetic during their medical education.

## Competing interests

All authors (TAQ, RAP, DFW and JAB) declare that they have no competing interest: no support from any organisations, no financial relationships with any organisations and no other relationships or activities that could influence the submitted work.

## Authors' contributions

TAQ participated in the conception, design and coordination of the study, the statistical analysis and drafted the manuscript. JAB and DFW participated in the conception and design of the study and helped to draft the manuscript. RAP performed the statistical analysis and helped to draft the manuscript. All authors (TAQ, RAP, DFW and JAB) declare that they have read and approved the final manuscript.

## Pre-publication history

The pre-publication history for this paper can be accessed here:

http://www.biomedcentral.com/1472-6920/11/90/prepub
